# Specific Forms of Graphene Quantum Dots Induce Apoptosis and Cell Cycle Arrest in Breast Cancer Cells

**DOI:** 10.3390/ijms24044046

**Published:** 2023-02-17

**Authors:** Tien-Hsiung Ku, Wen-Ting Shen, Chien-Te Hsieh, Grace Shiahuy Chen, Wei-Chung Shia

**Affiliations:** 1 Department of Anesthesiology, Changhua Christian Hospital, Changhua 50050, Taiwan; 2 Artificial Intelligence Development Center, Changhua Christian Hospital, Changhua 50050, Taiwan; 3 Department of Applied Chemistry, Providence University, Taichung 43301, Taiwan; 4 Molecular Medicine Laboratory, Department of Research, Changhua Christian Hospital, Changhua 50050, Taiwan; 5 Department of Chemical Engineering and Materials Science, Yuan Ze University, Taoyuan 32003, Taiwan

**Keywords:** graphene quantum dots, biomedicine, ER+ breast cancer, apoptosis

## Abstract

Graphene quantum dots (GQDs), nanomaterials derived from graphene and carbon dots, are highly stable, soluble, and have exceptional optical properties. Further, they have low toxicity and are excellent vehicles for carrying drugs or fluorescein dyes. Specific forms of GQDs can induce apoptosis and could be used to treat cancers. In this study, three forms of GQDs (GQD (nitrogen:carbon = 1:3), ortho-GQD, and meta-GQD) were screened and tested for their potential to inhibit breast cancer cell (MCF-7, BT-474, MDA-MB-231, and T-47D) growth. All three GQDs decreased cell viability after 72 h of treatment and specifically affected breast cancer cell proliferation. An assay for the expression of apoptotic proteins revealed that p21 and p27 were up-regulated (1.41-fold and 4.75-fold) after treatment. In particular, ortho-GQD-treated cells showed G2/M phase arrest. The GQDs specifically induced apoptosis in estrogen receptor-positive breast cancer cell lines. These results indicate that these GQDs induce apoptosis and G2/M cell cycle arrest in specific breast cancer subtypes and could potentially be used for treating breast cancers.

## 1. Introduction

Graphene quantum dots (GQDs) have attracted extensive attention in the biomedical field owing to their excellent physicochemical properties, including excellent solubility, high stability, and low toxicity [1]. Owing to their stable and high photoluminescence, GQDs are extensively used as luminophore particles or dyes. Hydrothermal synthetic methods have been developed for preparing polyethylene glycol (PEG)-passivated carbon nanostructures in the form of carbon nanodots [2]. A one-pot solvothermal synthesis for PEG-coated GQD clusters that generate green and red fluorescence under blue-light irradiation has been proposed [3]. Such GQDs may be excellent materials for applying fluorescent dyes to cells or blood vessels.

The potential hazards of GQDs to humans and other biological systems must be carefully investigated to enable their biomedical applications. Some carbon nanomaterials, including carbon nanotubes and nanodiamonds, cause DNA damage, although they do not exhibit severe cytotoxicity. The potential DNA damage (i.e., genotoxicity) by GQDs must be assessed because of the strong correlation between DNA damage and mutations or cancer. Recent studies on the in vitro and in vivo cytotoxicity of graphene-based materials have shown that GQD particles smaller than a certain threshold or concentration (up to 100 μg/mL) show no apparent toxicity in a range of cell lines [4,5,6,7]. Moreover, GQDs passivated with PEG derivatives generate reactive oxygen species (ROS) upon light irradiation [8], which can cause potential DNA damage. To date, no genotoxicity studies on GQDs have been reported. Cytotoxicity studies at the cellular level are warranted to develop GQDs for clinical use.

At the same time, several studies have also attempted to apply the cytotoxic properties of GQDs to treating cancers. Additionally, the physical properties of GQDs can induce several cellular mechanisms and have attracted attention as potential therapeutic compounds. In terms of breast cancer, (1) GQDs’ characteristics, such as water solubility, chemical inertness, high biocompatibility, and low toxicity, make them excellent drug delivery systems [9,10]; (2) the fluorescence and self-luminous properties of GQDs allow simultaneous detection [11] and treatment of cancer cells (also known as photodynamic therapy) [8,12]; and (3) the high DNA breakdown and increased aggregation in the nucleus of GQDs enhance the effect of chemotherapeutic drugs acting on the nucleus [13]. Although several studies have explored the effect of the physical properties of GQDs on breast cancer, studies on GQD cytotoxicity and the effects on apoptosis/cell cycle regulation of specific molecular subtypes of cancer are less common. In our previous study, we successfully synthesized a series of PEG-coated GQD clusters using the one-pot hydrothermal route and simultaneously validated their luminescent properties [14]. During cell experiments, we found that some isomers of these GQDs can affect the growth of specific subtypes of breast cancer cell lines by inducing apoptosis. In this study, we report the preliminary result and investigate whether these specific GQDs reduce the viability of breast cancer cell lines by triggering apoptosis.

## 2. Results

### 2.1. Screening GQDs Triggering Apoptosis

**Figure 1:**
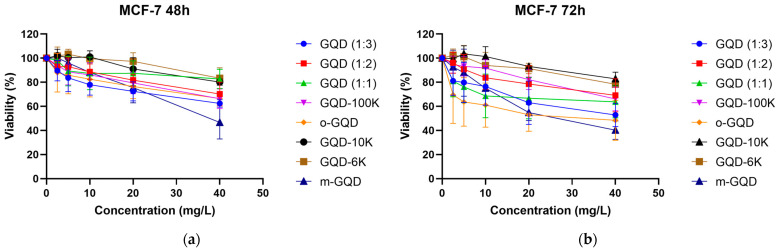
Changes in cell viability of MCF-7 cells treated with different doses of eight GQDs for (**a**) 48 h and (**b**) 72 h. The control group comprises cells without GQDs treatment (0 mg/L). The data indicate averages from triplicates, and the error bars indicate standard deviation (SD). Significance is determined by using two-way ANOVA statistical analysis.

We treated MCF-7 cells with different doses (2.5/5/10/20/40 mg/L) of the eight GQD clusters for 48 h or 72 h and determined the cell viability using an MTT assay. The control group comprised the cells without GQDs treatment (0 mg/L). MCF-7 is an invasive breast ductal carcinoma (IDC) cell line expressing estrogen receptors (ERs)/progesterone receptors (PRs). Figure 1 shows the cell viability curves. Based on this, we identified three GQDs: GQD with a nitrogen and carbon ratio of 1:3 (GQD (1:3)), ortho-GQD (o-GQD), and meta-GQD (m-GQD), which remarkably reduced the viability of MCF-7 cells. The optimal doses of these GQDs were also determined.

The three GQDs identified in the screening, GQD (1:3), o-GQD, and m-GQD, significantly reduced the viability of MCF-7 breast cancer cells, with o-GQD inducing the most significant reduction in the viability of ER+ breast cancer cells (28.36% at 72 h post-treatment) (Figure 1). GQD (1:3) and m-GQD induced similar decreases in the viability of human epidermal growth factor receptor 2 (HER2)-positive breast cancer cells, showing 54.32% and 63.33% viabilities, respectively, after 72 h of treatment.

### 2.2. Effects of the Three GQDs on the Viability of ER+/TNBC Breast Cancer Cell Lines

**Figure 2:**
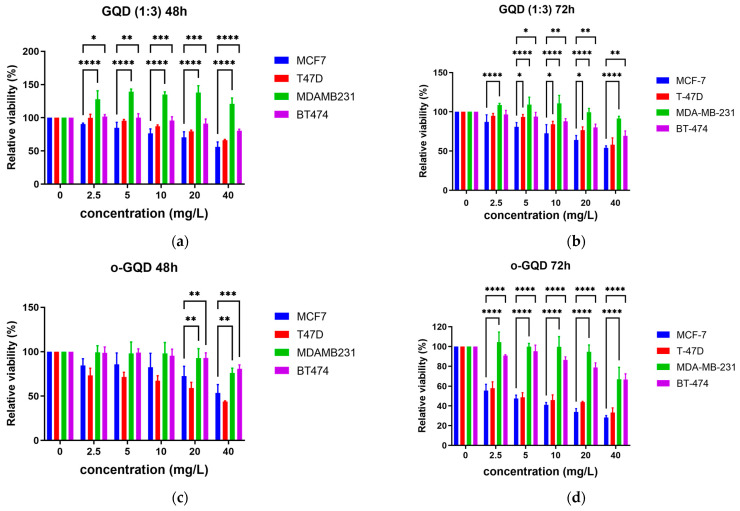

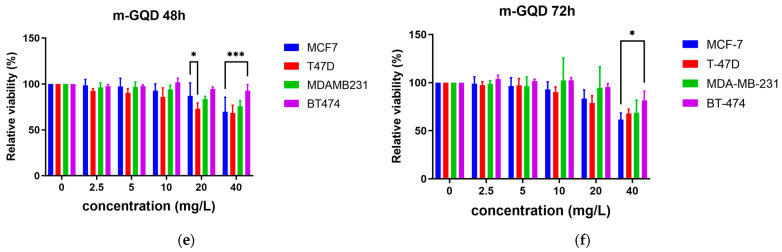
Relative cell viability of four breast cancer cell lines MCF-7, T-47D, MDA-MB-231, and BT-474 (**a**) treated with GQD (1:3) for 48 h; (**b**) treated with GQD (1:3) for 72 h; (**c**) treated with o-GQD for 48 h; (**d**) treated with o-GQD for 72 h; (**e**) treated with m-GQD for 48 h; and (**f**) treated with m-GQD for 72 h, as determined using MTT assay. The data indicate averages from triplicates, and the error bars indicate SD. Significance is determined by using two-way ANOVA statistical analysis. The significant differences are indicated by using *. *: *p* < 0.05, **: *p* < 0.01, ***: *p* < 0.001, ****: *p* < 0.0001.

The effect of five doses (2.5/5/10/20/40 mg/L) of the three GQD clusters (o-GQD, m-GQD, and GQD (1:3)) on the viability of the ER+/PR+/HER2+ breast cancer cell line (BT-474), two ER+/HER2− breast cancer cell lines (MCF-7 and T-47D), and a triple-negative breast cancer (TNBC; ER–/PR–/HER2–) cell line (MDA-MB-231) was determined using an MTT assay (Figure 2). Cell viability at 48 h and 72 h post-treatment was determined. A significant decrease in the viability of ER+ breast cancer cell lines (MCF-7 and T-47D) was observed after treatment with the three GQD clusters, with the o-GQD cluster treatment showing the most significant decrease. In contrast, the TNBC and the ER+/PR+/HER2+ cell lines were less responsive to the GQDs.

MCF-7 and T-47D cells treated with the GQDs did not show a significant decrease in viability 48 h post-treatment. On the other hand, MDA-MB-231 and BT-474 cells treated with GQD (1:3) showed a significant decrease in cell viability 48 h post-treatment (concentrations from 5 to 40 mg/L, *p*-value from 4.6 × 10^−5^ to <1 × 10^−5^). Further, 48 h after o-GQD administration, MD-MB-231 and BT-474 treated with 20 to 40 mg/L GQDs showed significantly lower cell viabilities (*p*-value from 0.009 to 4 × 10^−5^) (Figure 2). MDA-MB-231 and BT-474 cells treated with o-GQD (concentrations from 2.5 to 40 mg/L) showed a significant reduction in cell viability 72 h post-treatment (*p* < 1 × 10^−5^) (Figure 2). The effect of GQD (1:3) on the cell viability of the MDA-MB-231 cell line was statistically significant at all tested concentrations (concentrations from 2.5 to 40 mg/L, *p* < 1 × 10^−5^), while BT-474 cells treated with GQD (1:3) showed a significant reduction in viability (*p* < 1 × 10^−5^) only at concentrations between 5 and 40 mg/L (Figure 2).

**Figure 3:**
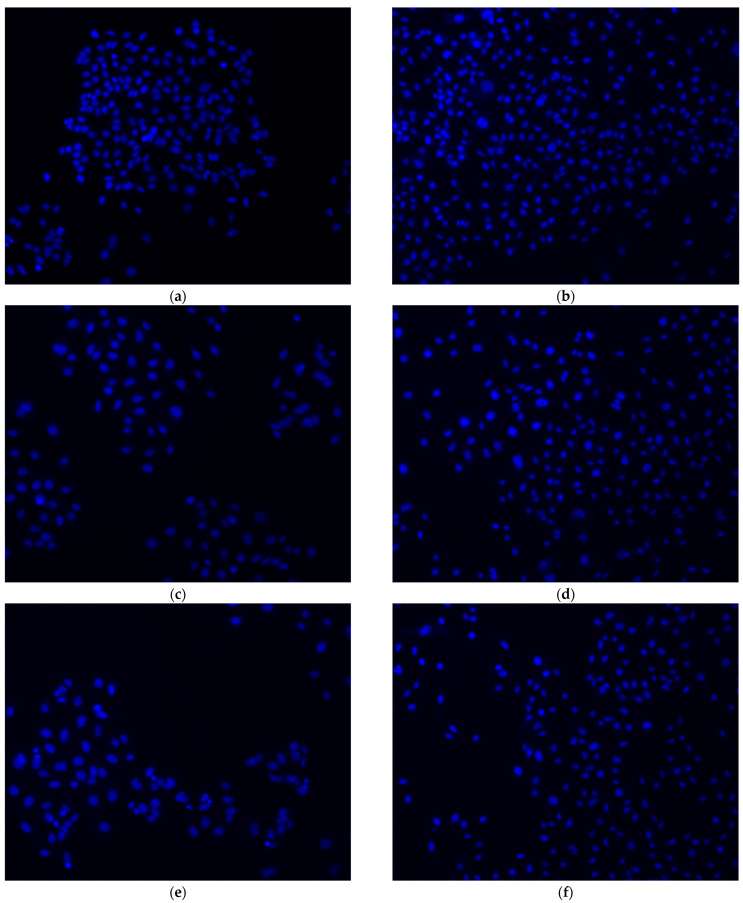
Representative micrographs showing nuclear morphology of MCF-7 (left column) and MDA-MB-231 (right column) cells after a 72 h treatment with (**a**,**b**) control (**c**,**d**) 2.5 mg/L, and (**e**,**f**) 10 mg/L o-GQD. Images are shown at 100× magnification. Images are shown at 200× magnification. The cell pointed by the white arrow is the cell where nuclear condensation and fragmentation occurs.

We stained the cells treated with o-GQD using Hoechst 33342 to observe changes in nuclear morphology and induction of apoptosis. We used both MCF-7 and MDA-MB-231 cell lines for comparison, and the results are shown in Figure 3. Cells treated with o-GQD showed nuclear condensation and fragmentation, indicating apoptosis. Further, an association was observed between the number of apoptotic cells, the concentration of o-GQD, and the molecular subtype of breast cancer.

Among the three GQDs, o-GQD showed a strong inhibitory effect on cell viability, while m-GQD showed the weakest effect. A two-way ANOVA analysis showed that o-GQD and GQD (1:3), at specific concentrations, had similar effects on the viability of specific breast cancer cells regardless of the treatment time (48 h and 72 h). Additionally, the effect of the GQDs was dependent on the cell line and the concentration (*p* < 1 × 10^−5^), and cell line dependency showed a greater effect (*p* < 1 × 10^−5^). Based on these, we used 2.5 and 10 mg/L as the test concentrations for the subsequent experiments.

### 2.3. o-GQD Treatment Triggers Apoptosis in ER+/TNBC Cell Lines

**Figure 4:**
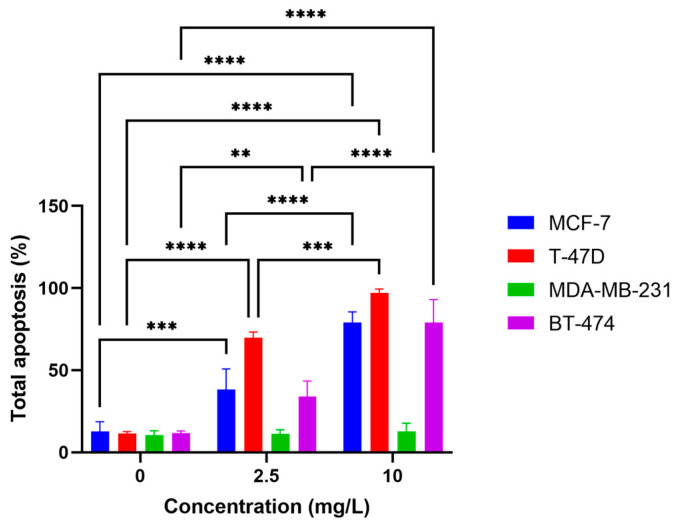
Apoptosis in the four breast cancer cell lines treated with o-GQD for 72 h determined using the Annexin V assay. The data indicate averages from triplicates, and the error bars indicate SD. Significance is determined by using two-way ANOVA statistical analysis. The significant differences are indicated by using *. **: *p* < 0.01, ***: *p* < 0.001, ****: *p* < 0.0001.

We used the human apoptosis antibody array and the Annexin V dead cell assay to confirm the induction of apoptosis after o-GQD treatment. Figure 4 shows the total apoptosis of the four breast cancer cell lines with or without 72 h o-GQD treatment, determined using the Annexing V assay. All four cell lines, including BT-474 and MDA-MB-231 cell lines—which displayed a less significant difference in previous experiments—and MCF-7 and T-47D cell lines, were treated with two different concentrations of o-GQD (2.5 and 10 mg/L). All cells showed early or late apoptosis, and T-47D displayed the highest apoptosis, with more than 85% of the cells undergoing apoptosis after treatments of o-GQD.

**Figure 5:**
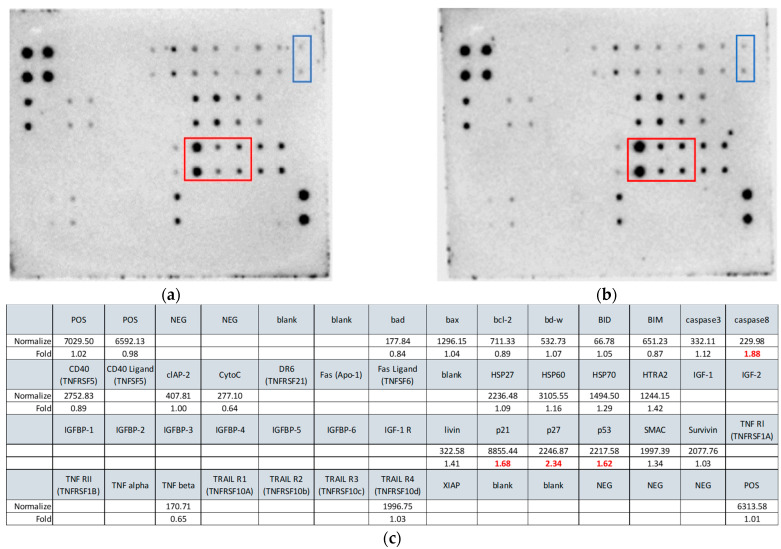
Expression of apoptosis-related proteins in the control and o-GQD-treated cells, measured using the apoptosis protein membrane array. Red box: (from left to right) p21, p27, and p53. Blue box: Caspase 8. (**a**) Control group cells; (**b**) o-GQD-treated cells (2.5 mg/L); (**c**) Apoptotic proteins and their expression ratios between pre-treated and post-treated cells. The position of the square in the figure and the protein name and expression level listed in the square correspond to the protein position on the protein membrane array (black dots).

A protein membrane array was used to detect the expression of apoptosis-related proteins in untreated and o-GQD-treated cells (Figure 5). This experiment aimed to identify the possible molecular mechanisms triggered by o-GQD treatment by investigating the ratio of expression of apoptosis-related proteins and providing guidelines for follow-up investigations. The list of the apoptosis proteins detected in our samples and their relative expression compared to the control is provided in Figure 5c. A change in the expression by 1.5-fold between the pre-treated and post-treated cells was considered significant. Four proteins (p27, caspase 8, p21, and p53) met this criterion, and HTRA2 approached this condition. The ratios of expression of three positive control normalization (POS) points provided by the manufacturer between the untreated and treated cells were 1.02, 0.98, and 1.01, indicating the reliability of the results.

### 2.4. The Effect of o-GQD on Cell Cycle Arrest and Related Signaling Pathway

**Figure 6:**
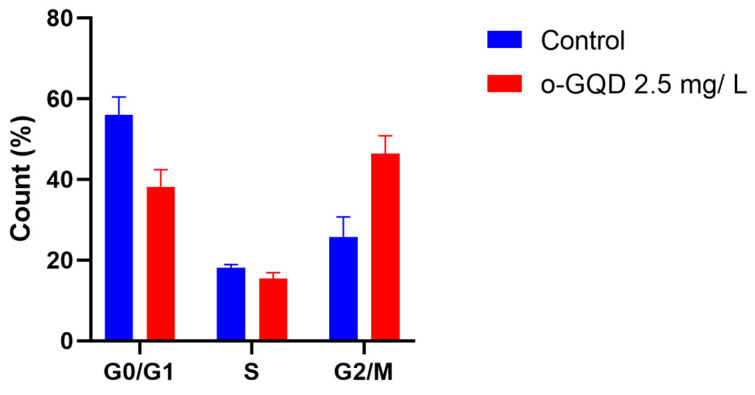
Percentage of cells in each cell cycle phase in MCF-7 cells with/without o-GQD (2.5 mg/L) treatment. The data indicate averages from triplicates, and the error bars indicate SD.

In the previous experiment, we confirmed that o-GQD-treated ER+ breast cancer cells showed higher expression of p21 and p27 compared to untreated cells. p21 and p27 belong to the kinase inhibitor protein (KIP) family. Further, the progression of the cell cycle is controlled by the cyclin-dependent kinases (CDKs) family and inhibited by CDK (CDK4 or INK4) and KIP inhibitors [15,16,17]. Therefore, we explored the effect of o-GQD on cell cycle arrest in the ER+ breast cancer cell line, MCF-7. MCF-7 cells treated with 2.5 mg/L o-GQD (experimental group) underwent G2/M cell cycle arrest, unlike the untreated cells (Figure 6). Statistical analysis showed that the average number of cells in the G0/G1 and G2/M phases significantly differed between the control group and the experimental group (G0/G1: *p* = 3 × 10^−5^; G2/M: *p* < 1 × 10^−5^). Previous studies have shown a similar up-regulation of p21/p27 protein expression in o-GQD-treated cells and a G2/M cell cycle arrest [18,19].

**Figure 7:**
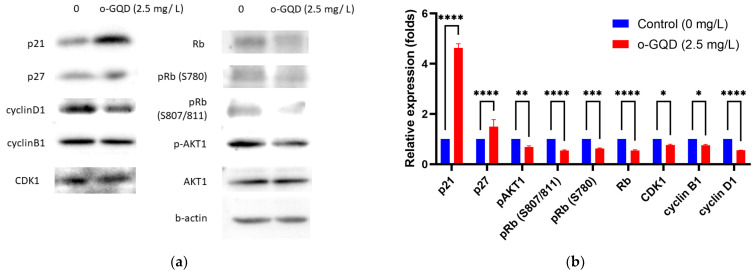
(**a**) Representative Western blot showing the expression of cell cycle-related proteins in MCF-7 cells treated with or without o-GQD for 72, (**b**) and their quantitative evaluation. The data indicate averages from triplicates, and the error bars indicate SD. Significance is determined by using two-way ANOVA statistical analysis. The significant differences are indicated by using *. *: *p* < 0.05, **: *p* < 0.01, ***: *p* < 0.001, ****: *p* < 0.0001.

We further verified the expression of cell cycle-related proteins, including phosphorylated retinoblastoma tumor suppressor protein (RB), protein kinase B (AKT), and CDK1, using Western blotting (Figure 7a,b). Western blot experiments were performed using MCF-7 cells treated with or without 2.5 mg/L o-GQD. The protein expression was also quantified for comparison (Figure 7b). The expression levels of p21 and p27 significantly increased with o-GQD treatment, in line with the results from the apoptosis protein array. No significant change in the expression of AKT was observed. However, a significant decrease in AKT1 phosphorylation (p-AKT1) was observed. Further, a marked reduction in related cyclins, including Rb, cyclin D1, cyclin B1, and CDK1, was observed in the treatment group. Quantitative analysis showed that the changes in protein expression between the groups were statistically significant (*p* < 0.001).

## 3. Discussion

Breast cancer, which is heterogeneous on the molecular level, is the most frequent malignancy in women worldwide. The molecular features include the activation of HER2, hormone receptors (ER and PR), and BRCA mutations [20]. Treatment strategies for breast cancer differ depending on the molecular subtype. In-depth research and understanding of the molecular mechanisms of breast cancer and related gene pathways in the past decade have resulted in the development of various new drugs for breast cancer. In this study, we used eight GQDs and confirmed their ability to reduce cell viability to a certain extent in four common breast cancer cell lines. The degree of reduction in cell viability varied among breast cancer cell subtypes. Among the GQDs tested, o-GQD showed the most significant reduction in cell viability. Our studies on apoptosis induction showed that the GQDs have significant cytotoxic effects on specific breast cancer subtypes.

**Figure 8:**
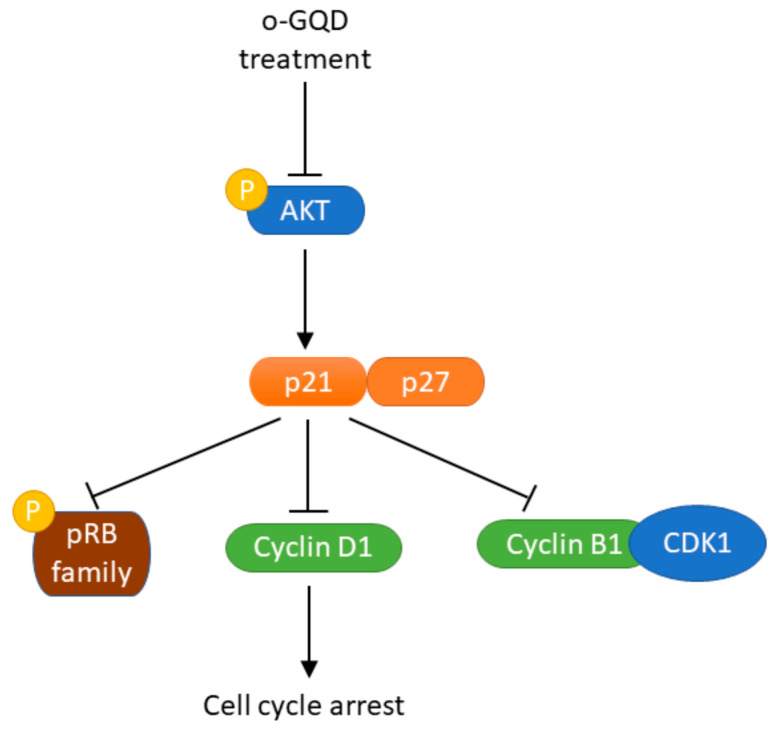
Schematic showing how o-GQD affected breast cancer cell signaling and triggered cell cycle arrest and apoptosis.

Our cell cycle assay revealed that o-GQD induced significant differences in breast cancer cells; therefore, we further analyzed cell cycle-related proteins. Western blot analysis showed that p21 and p27 expression was significantly increased after o-GQD administration, which was consistent with the results from the apoptosis protein array. p21 and p27 affect the cell cycle by regulating the expression of CDKs and cyclin [19], and previous studies also indicated that the increase of p21 and p21 protein expression promotes G2/M cell cycle arrest [21,22]. Our results also revealed that o-GQD treatment down-regulated cyclin D1 and cyclin B1 expression. o-GQD inhibits cell cycle-related proteins by stimulating the expression of p21 and p27, resulting in cell cycle arrest. Our cell cycle experiments indicated that o-GQDs lead to a G2/M phase arrest of the cell cycle (related results are shown in Figure 5). CDK1 and cyclin B1, mainly expressed in the G2/M phase, were significantly reduced in the cells treated with o-GQD, as demonstrated by our Western blot experiment. For the pRb protein, when the phosphorylation of Rb is inhibited, cell cycle-related genes cannot be successfully transcribed, resulting in cell cycle arrest. Although it is generally believed that the Rb protein regulated by p21 in the cell cycle mainly affects the cell cycle arrest in the G1 phase, there are also other literature results that show that when cells undergo G2/M cell cycle arrest, the expression of pRb protein will also decrease at the same time [23,24]. A graphical representation of the possible mechanism by which o-GQD treatment induces cell cycle arrest and apoptosis in breast cancer cells is shown in Figure 8.

We used a panel of four breast cancer cell lines, which included HER2+ and TNBC lines, to verify the effect of GODs on apoptosis and cell cycle progression. Our results showed that the GQDs significantly affected the proliferation of ER+ breast cancer cell lines. The PI3K/AKT/mTOR signaling pathway is one of the most important molecular signaling pathways that modulate proliferation, survival, invasion, migration, apoptosis, glucose metabolism, and DNA repair in ER+ breast cancer cells [25]. We showed that o-GQD inhibited the phosphorylation of AKT, suggesting that o-GQD affects the PI3K/AKT/mTOR signaling pathway, reduces p21 inhibition by inhibiting AKT phosphorylation, and increases the expression of p21, thereby inhibiting the expression of cell cycle-related proteins and triggering cell cycle arrest and, eventually, apoptosis.

This study still has some limitations which need to be clarified and enhanced in future follow-up studies. First, the detailed mechanisms underlying o-GQD-triggered apoptosis in ER+ cancer cell lines, including whether it acts inside or outside the cell or affects a specific cell organelle, need to be identified. Previous studies have reported that the most common mechanisms underlying graphene-triggered apoptosis were DNA damage and mitochondrial damage caused by ROS or oxidative stress [26,27,28]. Our results from the apoptosis protein array showed a slight up-regulation of the mitochondrial apoptosis-promoting proteins, including the second mitochondria-derived activator of caspase (SMAC; 1.34-fold) and the upstream protein p53 (TP53; 1.62-fold). However, subsequent studies did not show significant mitochondrial damage 72 h after the administration of o-GQD (detailed result and method shown in Appendix A). DNA damage-related studies were not performed due to time and technical constraints. Future studies should determine the effect of o-GQD treatment on breast cancer cell DNA.

Our preliminary experiments showed that o-GQD does not trigger apoptosis in a nontumorigenic human breast epithelial cell line (detailed materials, method, and result shown in Appendix B). Whether o-GQD inhibits the estrogen signaling pathway should also be evaluated. Owing to the high complexity of the signaling mechanisms and molecular pathways related to ER+ cell apoptosis and cell cycle arrest, they were not the primary focus of this study and were not investigated in depth. The signaling pathways in ER+ cancers affected by GQD treatment require further investigation by transcriptome expression analysis and next-generation sequencing. This is a major limitation of the current study and a direction for our future work.

In conclusion, our findings provide a preliminary understanding of the effect of GQDs on apoptosis and cell cycle progression. Further pre-clinical and clinical studies on GQDs are warranted to develop them as therapeutic drugs for breast cancer in the clinic. Owing to the complexity of ER-related regulatory pathways, this study did not fully explore the selective cell viability inhibition of GQD on ER+ cells. In the future, we will investigate the molecular and biological properties of the GQDs in-depth.

## 4. Materials and Methods

### 4.1. Reagents

A detailed procedure of a one-pot hydrothermal route for synthesizing PEG-coated GQD clusters used in this study has been described and published previously [14]. Briefly, o-phenylenediamine (o-PD) and PEG were first homogenously dispersed in 50 mL of deionized water and thoroughly stirred to prepare a well-mixed solution. Second, the suspension was placed into an autoclave, and hydrothermal synthesis was conducted at 300 °C for ~120 min. After cooling to the ambient temperature, the prepared suspension with PEG-coated GQD samples was freeze-dried at −30 °C for 72 h. The PEG-coated GQD samples were filtered using a microporous separator with an average pore size of ~0.02 μm to remove any insoluble residuals. The purified carbon nanodots (CNDs) were dispersed in ultrapure water before conducting any experiment. The whole hydrothermal synthesis and modification procedure of GQD was conducted and finished in the laboratory of co-authors (P.C.Y., C.T.H., and Y.X.D.). Eight PEG-coated GQD clusters with different structures or molecular weights were generated during the synthesis, depending on the ratio of nitrogen and carbon in their finished products. The stock concentration of all GQDs was 200 mg/L. The GQDs utilized in this study are shown in Table 1.

### 4.2. Cell Culture

T-47D, MCF-7, and MDA-MB-231 breast cancer cells were maintained in Dulbecco’s Modified Eagle Medium (DMEM; high glucose) (12800-017; Gibco, Waltham, MA, USA) containing 10% fetal bovine serum (FBS; VW-97068-085; VWR, Radnor, PA, USA), 10 mM HEPES buffer (15630-080; Gibco), and 50 μg/mL gentamicin (15750-060; Gibco). BT-474 cells were cultured in RPMI-1640 medium (SH-30027.01; Hyclone, Logan, UT, USA) with 10% FBS and 50 μg/mL gentamicin. All cells were cultured at 37 °C and 5% CO_2_.

### 4.3. Cell Viability Assay

MTT (3-(4,5-dimethylthiazol-2-yl)-2,5-diphenyltetrazolium bromide) was dissolved in phosphate-buffered saline (PBS), to a final concentration of 0.5 mg/mL. Cells (5000 cells/well) were seeded in a 96-well plate and treated with different GQDs. After 48 and 72 h, the samples were washed once with PBS. MTT was added into each well and incubated at 37 °C for 4 h. MTT was removed, DMSO was added to dissolve the formazan product, and the absorbance at 570 nm was measured by the EzDrop 1000 (Blue-Ray Biotech, New Taipei City, Taiwan).

### 4.4. Apoptosis Assay

To analyze apoptotic stages and cell death after GQD treatment, cells were seeded (8 × 10^4^/mL) in a 6 cm dish. After 72 h of treatment, cells were trypsinized, collected, and analyzed by flow cytometry using the Muse^®^ Annexin V and Dead Cell Assay kit (MCH100105; Luminex, Austin, TX, USA) and the Muse Cell Analyzer (Luminex, Austin, TX, USA), following the manufacturer’s protocols.

### 4.5. Cell Cycle Assay

Cells were seeded (8 × 10^4^/mL) in a 6 cm dish. After 72 h of treatment, cells were trypsinized, collected, and fixed with ice-cold 70% ethanol for 16 h at −20 °C. Cell cycle analysis was performed using the Muse^®^ Cell Cycle Kit and analyzed by the Muse Cell Analyzer (Luminex, Austin, TX, USA).

### 4.6. Apoptosis Antibody Array

The human apoptosis antibody array kit (AAH-APO-1; Ray Biotech, Peachtree Corners, GA, USA) was used to screen the apoptosis-related proteins. The final amount of protein was 200 μg.

### 4.7. Western Blotting

Cells were seeded (5 × 10^5^/mL) in 10 cm dishes. After 72 h of treatment, cells were trypsinized, collected, and lysed with RIPA lysis buffer (20-188; Millipore, Billerica, MA, USA) containing protease and phosphatase inhibitors (protease inhibitor cocktail set III, 535410; Millipore). After centrifugation at 16,000× *g* for 10 min at 4 °C in an Eppendorf Centrifuge 5418 R with angle rotor F-45-30-11 (Eppendorf Himac Technologies, Hamburg, Germany), the supernatant was collected. Protein concentrations were quantified using the bicinchoninic acid (BCA) assay (Dual-Range BCA Protein Assay Kit, BC03-500; Visual Protein, Taipei, Taiwan). Equal amounts of the protein samples were electrophoresed on a 12% or 8% SDS-PAGE and then transferred to a 0.45 μm PVDF membrane (IPVH00010; Millipore). The membranes were blocked with 5% skimmed milk (prepared with TBST buffer). The following primary antibodies were used: Rb (9309s; Cell Signaling Technology, Danvers, MA, USA), phospho-Rb (S780) (ab131264; Abcam, Cambridge, UK), phospho-Rb (S807/811) (9308s; Cell Signaling Technology), p21 (ab109520; Abcam), p27/KIP1 (ab32034; Abcam), ERK1/2 (A16686; Abclonal, Taipei, Taiwan), phosphor-ERK (AP1120; Abclonal), mTOR (A11354; Abclonal), phosphor-mTOR (S2248) (AP0094; Abclonal), cyclin D1 (33-3500; Invitrogen, Waltham, MA, USA), and CDK 4 (AHZ0202; Invitrogen), and beta-actin (MA515739; Invitrogen) was used as an internal control in this study. Secondary antibodies used were goat anti-rabbit IgG (H+L), HRP (32460; Invitrogen), and goat anti-mouse IgG (H+L), HRP (626520; Invitrogen). The density of bands was quantified using ImageJ software [29].

### 4.8. Hoechst Staining

Cells (2 × 10^4^/mL) were seeded in a 12-well plate. After 72 h of treatment, cells were washed with PBS and fixed with methanol at room temperature for 15 min. Hoechst 33342 dye (Invitrogen, H3570) was diluted with PBS to a final dilution of 1:10,000, and 1 mL of the staining solution was added to each well. After incubation at room temperature for 20 min, the wells were washed three times with PBS, and the images were procured using a fluorescence microscope.

### 4.9. Statistical Analysis

The mean values from triplicate experiments are shown. Two-way analysis of variance (ANOVA) followed by Dunnett’s multiple comparisons was used to compare multiple groups. A *p*-value < 0.05 was considered significant. All analyses were conducted using GraphPad Prism version 9.0 (GraphPad Software, San Diego, CA, USA).

## Figures and Tables

**Table 1:** 8 PEG-coated GQD clusters synthesized and utilized in this study.

No.	GQDs	Solvent
1	GQD (1:3) *	Water
2	GQD (1:2) *	Water
3	GQD (1:1) *	Water
4	GQD-100K	Water
5	o-GQD	Water
6	GQD-10K	Water
7	GQD-6K	Water
8	m-GQD	Water

* N-containing functional groups were distributed on the surface, imparting a stable aqueous dispersion of functionalized GQD suspension. N:C ratio.

**Figure A1:**
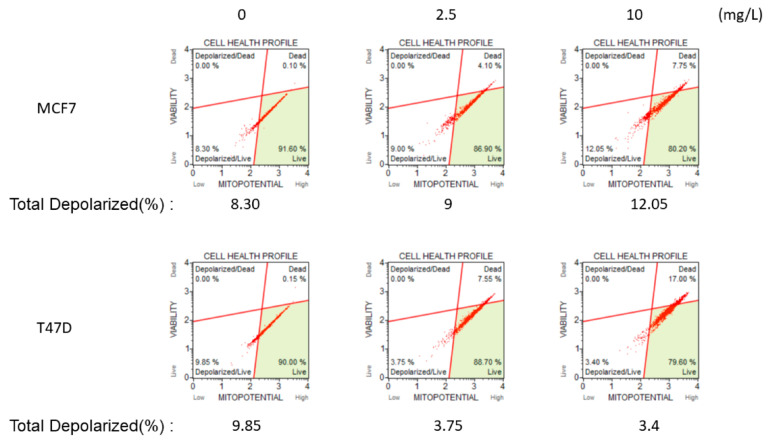
Using mitochondrial membrane potential experiment to observe the mitochondrial function of MCF-7 and T-47D breast cancer cell lines administrated with o-GQD for 72 h.

**Figure A2:**
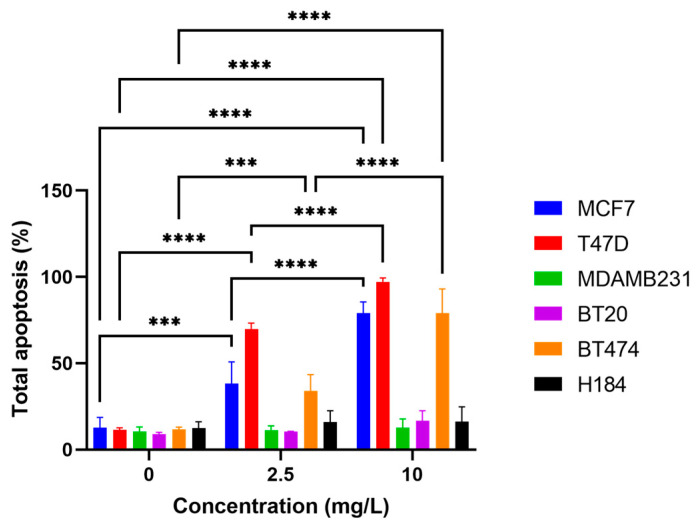
The cell viability of four breast cancer cell lines after 48 h and 72 h of specific GQD treatment. The data indicate averages from triplicates, and the error bars indicate SD. Significance is determined by using two-way ANOVA statistical analysis. The significant differences are indicated by using *. ***: *p* < 0.001, ****: *p* < 0.0001.

## Data Availability

Data are available from the corresponding author on reasonable request.

## Appendix A. Mitochondrial Membrane Potential Experiments of Breast Cancer Cell Lines after Administration of o-GQD

We used the mitochondrial membrane potential experiment to observe the mitochondrial function of two breast cancer cell lines (MCF-7 and T-47D) administrated with o-GQD for 72 h to confirm whether the reason for o-GQD reducing cell viability comes from mitochondrial functional damage. The effects of two doses (2.5/10 mg/L) were estimated. The control group comprises cells without o-GQD treatment (0 mg/L).

### Appendix A.1. Methods

Cells (8 × 104/mL) were seeded in a 6 cm dish; after 72 h of treatment, cells were trypsinized and collected, then measured with the Muse^®^ MitoPotential Kit (MCH100110) and analyzed by the Muse^®^ Cell Analyzer (Luminex, Austin, TX, USA).

### Appendix A.2. Results

The experimental results are shown in Figure A1. After comparing untreated and treated cells, the difference in total depolarization was small and did not reach statistical significance.

## Appendix B. Mitochondrial Membrane Potential Experiments of Breast Cancer Cell Lines after Administration of o-GQD

We provide the experimental results of cell viability experiments using the H184 cell line (normal breast epithelial cells) and a comparison with other breast cancer cell lines to confirm the cell toxicity of o-GQD to normal cells.

### Appendix B.1. Methods

MTT (3-(4,5-dimethylthiazol-2-yl)-2,5-diphenyltetrazolium bromide) was dissolved in phosphate-buffered saline (PBS), keeping 0.5 mg/mL as the final concentration. Cells (5000 cells/well) were seeded in a 96-well plate treated with different GQDs. After 48 and 72 h, the samples were washed once with PBS. MTT was added into each well and incubated at 37 °C for 4 h, then we removed the MTT and added DMSO to dissolve the formazan product. Absorbance was measured at 570 nm by the EzDrop 1000 (Blue-Ray Biotech, Taiwan).

### Appendix B.2. Results

It is initially shown that o-GQD has a certain degree of toxicity to normal mammary epithelial cells, but the decrease in cell viability is similar to that of non-ER+ cell lines (such as MDA-MB-231 or BT-20). The o-GQD only had a strong effect of reducing cell viability for ER+ cells strains. The quantitative results are presented in Figure A2.
